# Behavioral and EEG Measures Show no Amplifying Effects of Shared Attention on Attention or Memory

**DOI:** 10.1038/s41598-020-65311-7

**Published:** 2020-05-21

**Authors:** Noam Mairon, Mor Nahum, Arjen Stolk, Robert T. Knight, Anat Perry

**Affiliations:** 1 The Hebrew University of Jerusalem, Jerusalem, Israel; 2 University of California, Berkeley, CA USA

**Keywords:** Emotion, Social neuroscience, Human behaviour

## Abstract

Shared attention experiments examine the potential differences in function or behavior when stimuli are experienced alone or in the presence of others, and when simultaneous attention of the participants to the same stimulus or set is involved. Previous work has found enhanced reactions to emotional stimuli in social situations, yet these changes might represent enhanced communicative or motivational purposes. This study examines whether viewing emotional stimuli in the presence of another person influences attention to or memory for the stimulus. Participants passively viewed emotionally-valenced stimuli while completing another task (counting flowers). Each participant performed this task both alone and in a shared attention condition (simultaneously with another person in the same room) while EEG signals were measured. Recognition of the emotional pictures was later measured. A significant shared attention behavioral effect was found in the attention task but not in the recognition task. Compared to event-related potential responses for neutral pictures, we found higher P3b response for task relevant stimuli (flowers), and higher Late Positive Potential (LPP) responses for emotional stimuli. However, no main effect was found for shared attention between presence conditions. To conclude, shared attention may therefore have a more limited effect on cognitive processes than previously suggested.

## Introduction

Humans are social animals that often prefer acting together, rather than alone^1,2^. Potential differences in function or behavior when experienced alone or in the presence of others are referred to as co-presence effects that can be either generated from the mere presence of another or from a shared experience that occurs in the physical or psychological presence of another person^3–5^. When simultaneous attention of the participants to the same stimulus or set is involved, shared experience overlaps with the term shared attention^4,6^. Indeed, both shared experience and shared attention have been found to affect cognitive function and behavior, such as attention to a target stimuli^3,7^, social learning^8^, memory^9,10^, motivation^11^, judgment^12^ and inhibitory control^13^. Specifically, shared attention has been found to amplify these processes (etc. memories, emotions, and behavioral learning)^4,6^. For example, higher manifestation of social learning was found in the shared attention condition compared to an unshared condition^8^.

Several^5,14,15^ studies have examined the effect of shared attention in emotional contexts, but yielded mixed results. For example, Shteynberg and colleagues examined online shared attention effects on reactions to emotional stimuli. In a series of studies, they presented either short clips or pictures to participants and found enhancement of the subjective feelings (self-reported) for emotional stimuli in the online presence of another person. Specifically, scary advertisements led to higher “scariness” scores in the shared attention condition compared to the same stimuli viewed alone. Similarly, in response to positively and negatively-valenced pictures (the International Affective Picture System; IAPS^16^), self-reported feelings were magnified in the direction of the attended object’s valence in the shared attention condition. Such effects were not found for neutrally-valenced images^12^. Similarly, in an fMRI study by Wagner *et al*.^5^, participants believed that in some of the trials a friend was also viewing the same stimuli (IAPS pictures). The authors found increased fMRI activation in the brain reward system and in prefrontal areas such as the dorsolateral prefrontal cortex (DLPFC) for the shared viewing condition. The authors suggested that shared emotional experience is rewarded by brain activation that strengthens a motivational tendency to bond and affiliate with others in emotional situations^5^.

Along the same lines, Boothby, Clark, & Bargh examined the effect of shared attention in a simple chocolate tasting test, by having participants taste the chocolate either simultaneously with another participant (“shared” condition) or alone yet in the presence of another participant (“unshared” condition). Their results show that shared attention magnifies the perceived pleasantness/ unpleasantness taste of a chocolate. These results led the authors to suggest the Emotional Amplification Theory, according to which experiences are amplified when shared and mediated by presentation of the stimuli in the shared condition^6^.

However, other studies examining the effect of shared experience have not been consistent with this theoretical concept^17,18^. For example, Fridlund^18^ found that positively-valenced stimuli elicited smiling in participants that varied monotonically with the perceived sociality of the shared experience conditions (shared experience, audience or alone); however, this effect was not related to the subjective emotion reported by the participants. Another study, which measured face expressions and subjective emotions of participants while they watched a sad movie alone or in a shared condition, found changes in the frequency of sad expressions between presence conditions; however, there was no relation between facial expressions and the reported subjective feelings^17^.

Recently, Jolly *et al*.^14^ suggested that such behavioral changes represent enhanced communicative or motivational behavior rather than an elevated subjective experience in co-presence situations. In a series of eight experiments, they failed to find an effect of shared attention on participants’ emotional experience while they watched emotional video clips, although participants seemed to value shared experiences and were motivated to engage in them. This result remained constant among various experimental setups, which manipulated participants’ physical co-presence or shared experience^14^.

Nonetheless, it seems that shared attention can alter memory for stimuli^19^. For example, several studies examined the effect of shared attention on memory, and showed higher accuracy rates as well as shorter response times in recognizing stimuli presented in the shared condition^10,20^. Furthermore, several studies showed that performing a task with a joint setup (having stimuli assigned to each participant from a different category or type) enhances attention to stimuli from the partners’ category more than other task-irrelevant stimuli, in addition to stimuli of ones’ own category^7,9,21^. This effect was found in accordance to the psychological distance between participants – there was a larger shared-attention effect for participants that saw stimuli on the same screen than those who saw them separately^20^.

These mixed results call for a better understating of the effect of shared attention on one’s cognition and behavior. Moreover, while previous studies investigated the effects of shared attention on the subjective emotional reaction, none of these studies address the potential effect of shared attention on cognitive processing, and specifically on attention and memory for emotional stimuli. Furthermore, examining shared attention in a physical co-presence setup, using an objective measure, rather than a subjective self-report may provide a more accurate estimation of the shared attention effect.

To this end, the current study examined the effect of shared attention on attention to and memory for emotional stimuli (IAPS pictures;^16^), in a set-up using both behavioral measures and a dual-electroencephalogram (EEG) recording. Participants were first asked to count rare stimuli (pictures of flowers) that were interspersed among emotionally-valenced IAPS pictures with either positive, negative or neutral valence. The use of the IAPS pictures enables one to control for both the valence and the arousal of stimuli and counterbalance them between experimental sets^16,22,23^. Each participant performed this task twice: alone and simultaneously with another person in the room (shared condition), while EEG was recorded.

It has been previously suggested that shared attention may alter the underlying cognitive resources^4,5^. Although behavioral outcomes alone can be informative in examining the question of whether shared attention enhances attention and memory to emotionally valenced stimuli, it is limited to the participant’s explicit report (or sometimes reaction time measures). In the current study, EEG was used to examine the potential implicit effect of shared attention on attention to emotional stimuli. EEG allows for precise temporal resolution that can reveal differences between early and late processing of emotional stimuli^24^, which can complement behavioral measures.

For this purpose, we analyzed two Event-Related Potential (ERP) components time-locked to the presentation of stimuli during the flower-counting task: the Late Positive Potential (LPP) and P3b. The LPP is a positive deflection in EEG amplitude elicited by visual stimulus perception. The LPP typically arises over parietal sites, occurs 400–800 ms post-stimulus presentation, and is enhanced for emotionally salient relative to neutral stimuli^23,25–27^. The P3b, a positive deflection with maximal amplitude over centro-parietal scalp electrodes around 350-600 ms post-stimulus, is correlated with attention to task-related rare, yet anticipated, stimuli^28–30^. The P3b was calculated for flower stimuli and the LPP was calculated for emotionally-valenced stimuli, while neutral-valenced stimuli used as a control condition for both.

In addition, mu rhythms (8–13 Hz) are established EEG components associated with social attention, acquired using EEG in Centro-parietal areas^31–33^, and thus constitute a useful neural measure for the brain systems implicated in shared attention. When executing or observing actions, sensorimotor mu rhythms show a desynchronization of activity, as reflected in suppressed power relative to pre-action levels^34–36^. Moreover, greater mu suppression is found in social compared to non-social contexts, such as when one is participating in a social interaction^37,38^, deciphering the intentions of others from biological motion^39,40^, and understanding facial expressions^41^, or levels of pain^36^ of others. Note that apart from mu suppression, alpha suppression, measured in the same frequency range, but over parietal-occipital areas has been repeatedly shown to be affected by visual attention^31,36,42–44^. Both alpha and mu suppression can serve as useful measures of neural changes occurring as a result of shared attention.

We hypothesized that attention to and memory for stimuli will be magnified in the shared condition, leading to higher accuracy rates in the flower counting task (enhanced attention to the target stimuli) and to better performance in the recognition task (indirectly linked with attention to emotional stimuli during the counting flowers task). In addition, we predicted heightened ERP responses, manifested as increased positivity over parietal electrodes in response to targets (P3b) and to emotional IAPS stimuli (LPP). Lastly, in line with previous studies we expected to find stronger alpha suppression in the shared condition, indicating increased attentional processing^45^ as well as stronger mu suppression, indicating more social involvement, as was previously found in response to social context^37,38^.

## Results

### Flower-counting Task: Attention to Target Stimuli

#### Behavioral Differences between Conditions

**Figure 1:**
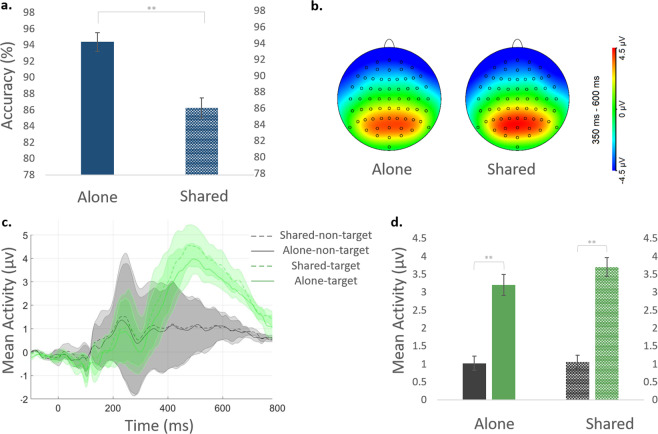
Performance on the flower counting task. (**a**) Accuracy rates on the flower counting task and; (**b**) P3b topography (µv) in the window of [350:600]ms after the presentation of target stimuli in the alone (left) and shared (right) viewing conditions; (**c**) Stimulus-locked average activity (in µv) in the time window of [−100:800]ms. ERP data was calculated separately for the two viewing conditions (alone, solid lines; shared, dashed lines) and for the different stimulus types (target - flowers, green; non-target - neutral IAPS, black); Shaded error regions represent standard deviation errors (using the ShadedErrorBar MATLAB function). ERPs were computed across Pz CPz POz; (**d**) P3b mean activity in the 350–600 ms window, for each condition: target (flowers, green) and non-target (neutral IAPS, black) stimuli, viewed alone (left) and under shared attention (right; dashed) conditions. Error bars represent standard error of mean (SEM).

A significant difference in flower counting was found in accuracy rates between the two conditions (alone/shared), such that accuracy was higher when the task was performed alone [N = 40*; M* ± *SD*; alone = 94.35 ± 7.5, shared = 86.25 ± 7.9; *t*(39) = 5.62, *p* = 0.00, *d* = 1.04, BF_10_ = 10254.28]) (Fig. 1a).

#### P3b

To examine differences in responses to target stimuli (flowers), we compared responses to target images with responses to neural IAPS images, by conducting a 2×2 ANOVA [condition (alone, shared) × stimulus type (target (flowers), non-target (neutral IAPS)], and a comparable Bayesian ANOVA. There were no statistically significant differences between the two conditions [N = 38*; M* ± *SD* (µv); 2.1 ± 0.21, 2.38 ± 0.18 for alone and shared, respectively; *F*(1,37) = 2.45, *p* = 0.126, *η*_*p*_^2^ = 0.06, BF_10_ = 0.27]. However, there was a significant main effect for stimulus type, revealing the expected larger P3b response to target compared to non-target stimuli [target = 3.44 ± 0.24, non-target = 1.05 ± 0.18; *F*(1,37) = 100.98, *p* = 0.00, *ηp*^2^ = 0.73, BF_10_ = 2.16 ×10^20^]. The interaction between condition and stimulus type was not significant [*F*(1,37) = 3.1, *p* = 0.087, *ηp*^2^ = 0.07, BF_10_ = 0.44] (Fig. 1b,c).

A permutation analysis which was conducted in order to control for the difference in the number of segments for each stimulus type yielded similar results for condition [*F*(1,37) = 0.71, *p* = 0.40, CI_F_ [0.18, 2.93]], stimulus type [*F*(1,37) = 71, *p* = 0.00, CI_F_ [60.52, 88.19]] and interaction [*F*(1,37) = 0.03, *p* = 0.85, CI_F_ [0, 2.56]].

### Flower-counting Task: Attention to Emotional Stimuli

#### LPP

**Figure 2:**
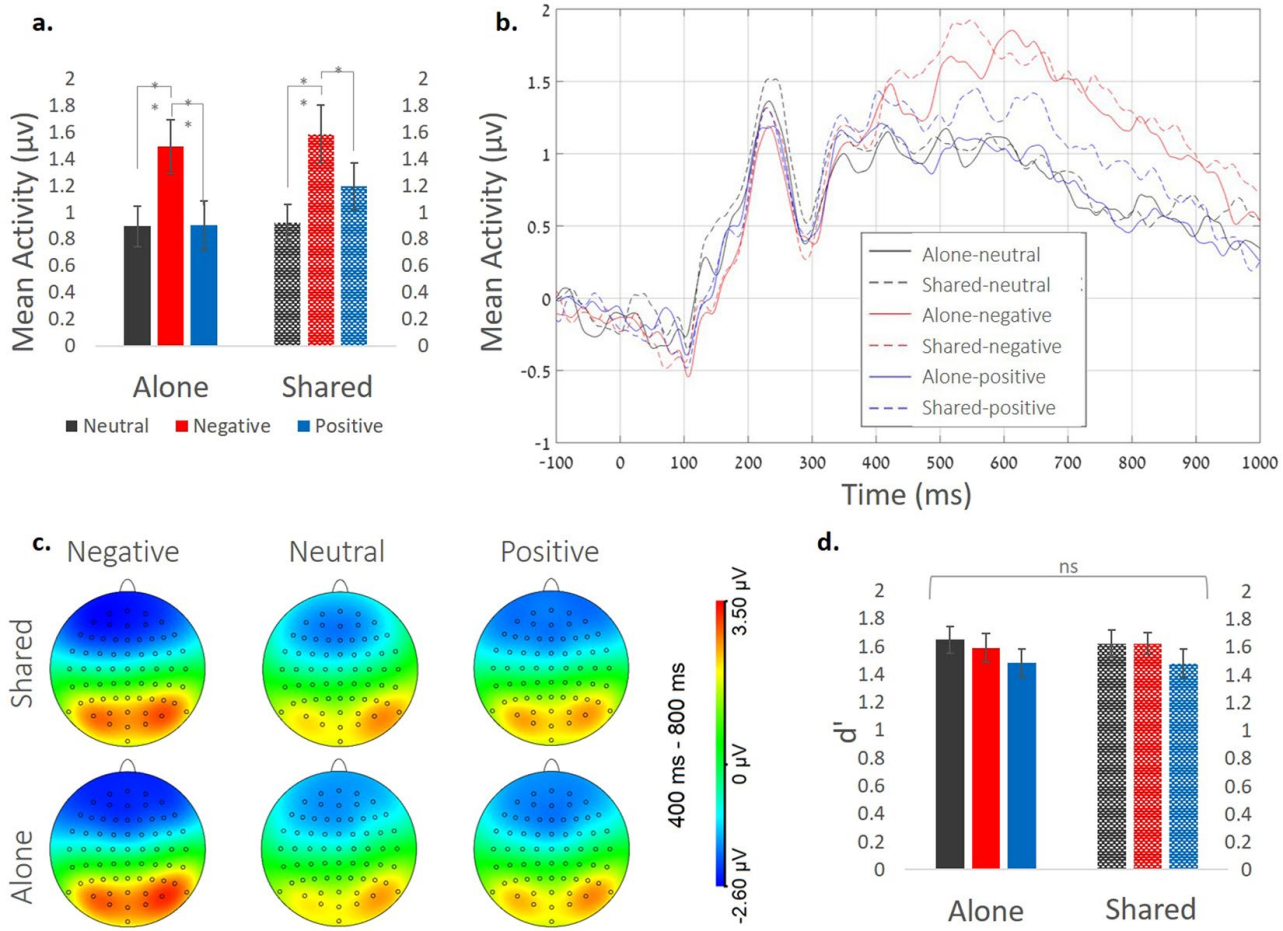
Shared attention effects for non-target emotional stimuli. On the flower counting task: (**a**) Mean LPP activity (in µv) in the time window of [400–800]ms averaged across the 3 electrode locations: POz, Pz and CPz; (**b**) Mean ERP activity in the time window of [−100:1000]ms (stimulus-locked) for POz, Pz and CPz ^*^; (**c**); LPP topography (µv) in the [400–800]ms window following the presentation of non-target stimuli in each condition, and; on the memory task: (**d**) Performance sensitivity (d’) for the recognition task. Error bars represent standard error of mean (SEM). *For shaded error regions see Supplementary Fig. 1.

We conducted a 2×3 ANOVA [condition × valence (neutral/negative/positive)] for the LPP amplitudes recorded during the flower counting task, as well as a comparable Bayesian ANOVA. We found a significant valence effect, reflecting increased neural LPP responses for stimuli with negative (low valence) compared to positive (high valence) or neutral images [N = 38*;* ERP amplitude: *M* ± *SD* (µv); neutral = 0.92 ± 0.14, negative = 1.54 ± 0.2, positive = 1.05 ± 0.18; *F*(1.56,57.78) = 19.18, *p* = 0.00, *ηp*^2^ = 0.34, BF_10_ = 2.27 × 10^7^]. However, there was no significant effect of condition [*M* ± *SD* (µv); alone = 1.1 ± 0.17, shared = 1.23 ± 0.17; *F*(1,37) = 1.77, *p* = 0.19, *ηp*^2^ = 0.046, BF_10_ = 0.39]. Additionally, the interaction between condition and valence was not significant [*F*(1.96,73.12) = 2.44, *p* = 0.09, *ηp*^2^ = 0.06, BF_10_ = 0.2] (Fig. 2a). We also calculated LPP mean activity in a posterior-parietal electrode composition (C1, C2, CP1, CP2, Cz, CPz/Pz) and a control analysis in frontal **sites** (Fpz, Fp1, Fp2). In both cases we got similar results to the first analysis (see Supplementary Information).

#### Alpha and Mu Rhythms

**Figure 3:**
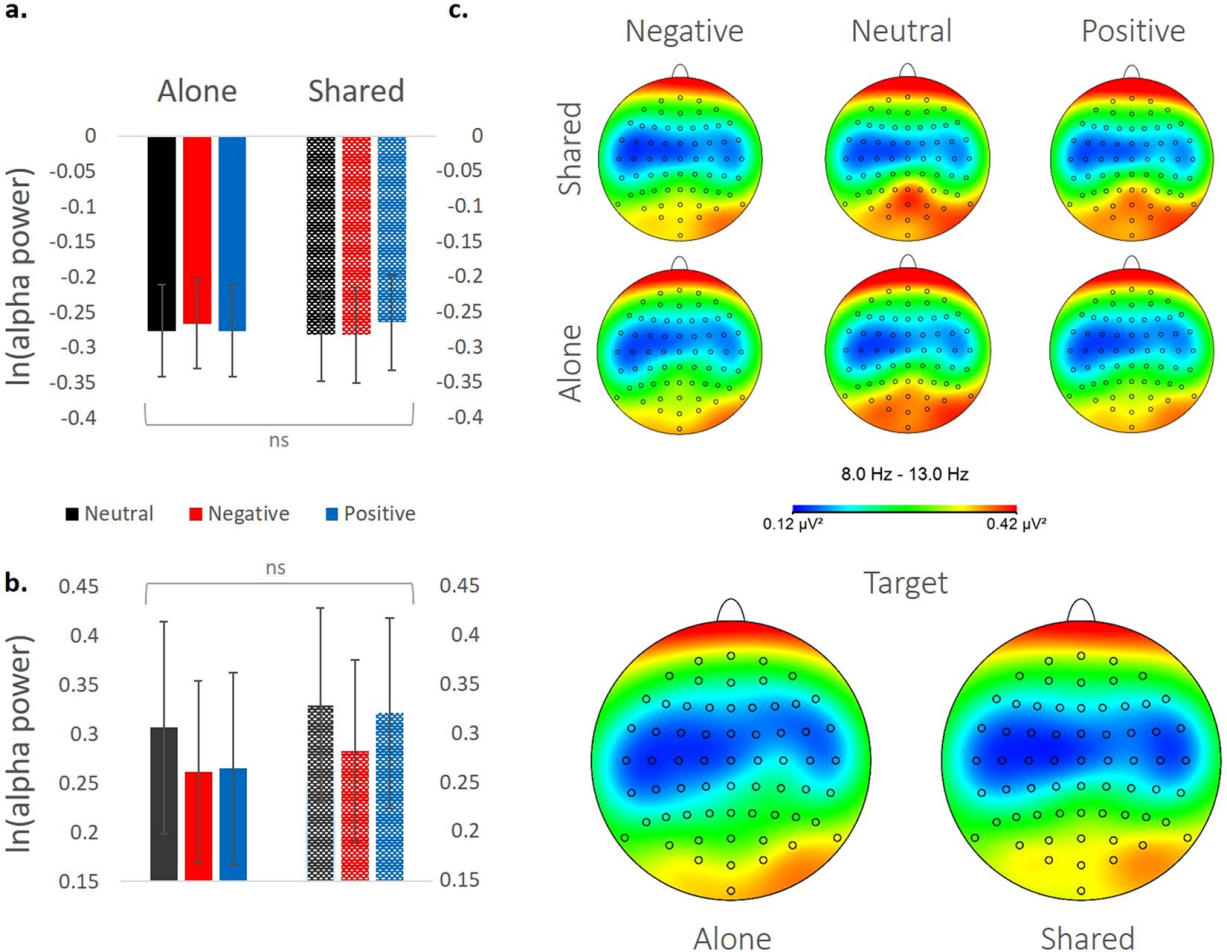
Alpha/mu band power (8Hz-13hz) during the flower counting task. (**a-b**) Logarithmic scale for the averaged activity within the 8–13 Hz range in locations C3, C4 (mu activity, panel (**a**), and in locations O1 O2 and Oz (alpha activity, panel (**b**). Error bars represent standard error of mean (SEM). (**c**) Topographic maps of power amplitude (µv^2^) in the 8–13 Hz range.

As alpha band power tends to differ significantly between participants^36,46^, we first normalized the data using a logarithmic scale (ln(mean activity)). We analyzed mu activity (8–13 Hz) over central areas (electrodes C3 and C4) as a measure of social attendance^31^ (Fig. 3a). There was no significant effect of condition on mu activity, confirmed via both ANOVA and Bayesian ANOVA [N = 38; alone = −0.27 ± 0.06, shared = −0.27 ± 0.06; *F* < 0.01, *p* = 0.96, *ηp*^2^ < 0.001, BF_10_ = 0.14]. The valence was also non-significant [neutral = −0.28 ± 0.06, negative = −0.275 ± 0.06, positive = −0.27 ± 0.06; *F*(1.93,74) = 1.22, *p* = 0.3, *ηp*^2^ = 0.03, BF_10_ = 0.05], as well as the condition X valence interaction [*F*(1.68,62.33) = 2.446, *p* = 0.1, *ηp*^2^ = 0.06, BF_10_ = 0.125].

Next, we analyzed alpha power (8–13 Hz) over occipital cortex (electrodes O1, O2 and Oz) as an additional measure of visual attention^42^ (Fig. 3c). There was no significant effect of condition on alpha rhythm, confirmed via both ANOVA and Bayesian ANOVA [N = 38; alone = 0.11 ± 0.04, shared = 0.135 ± 0.04; *F* < 1; *F*(1,37) = 0.76, *p* = 0.39, *ηp*^2^ = 0.02, BF_10_ = 0.64]. The valence effect was also non-significant [neutral = 0.125 ± 0.04, negative = 0.124 ± 0.04, positive = 0.124 ± 0.04; *F* < 1; *F*(1.77,65.7) = 0.003, *p* = 0.99, *ηp*^2^ < 0.001, BF_10_ = 0.045]. Although there was a significant condition X valence interaction [*F*(1.65,61.3) = 4.66, *p* = 0.018, *ηp*^2^ = 0.11], we found no support for it with Bayesian analysis accounts [BF_10_ = 0.2] nor simple effects between conditions (*p* > 0.05). As topographies hinted to a left laterality effect, we also computed a laterality index (C3-C4), and found no effect of shared attention in mu suppression activity [alone = −0.286 ± 0.11, shared = −0.253 ± 0.12; F(1,38) = 0.15, p = 0.69, ηp^2^ < 0.01] or valence [F(1.47,56.06) = 0.12, p = 0.88, ηp^2^ < 0.01] in this measure as well.

### Recognition Task: Memory for Emotional Stimuli

All subjects were better than chance in performing the task (i.e. had more than 55% success across conditions). To test whether shared attention manipulation (alone/shared) affected recognition of emotional pictures, we calculated d’ as a measure of recognition accuracy (see Methods). We found no main effect for condition [N = 42*; F* < 1; *F*(1,41) = 0.0, *p* = 1, *ηp*^2^ < 0.001, BF_10_ = 0.13] or for valence [neutral = 1.63 ± 0.87, negative = 1.6 ± 0.07, positive = 1.48 ± 0.09; *F*(1.94) = 2.47, *p* = 0.09, *ηp*^2^ = 0.05, BF_10_ = 0.61], nor a significant interaction between condition and valence [*F* < 1, *F*(1.78,73) = 0.23, *p* = 0.77, *ηp*^2^ < 0.001, BF_10_ = 0.08] (See Fig. 2d).

## Discussion

In the current study, we examined the effect of shared attention on attention to and recognition of emotional stimuli. Behaviorally, we found higher accuracy rates for the task when performed alone, compared to when it was performed in the presence of another person. At the neural level, we found no significant differences between conditions in either the P3b responses or alpha / mu band activity. We also found no differences in LPP responses to IAPS pictures. Lastly, we found no difference in recognition of emotional pictures as a function of shared attention.

Considering attention to the target stimuli first, the only solid shared attention effect found in our study was that of higher accuracy rates in the flower counting task when performed alone compared to when it was performed simultaneously with another person. This finding, although counter-intuitive, is supported by previous work suggesting that the shared condition may increase arousal^47^ or distract participants^48^, thus may impair performance compared to when the task is performed alone. Note that this effect was weakly reflected in the P3b neural response, showing the expected higher amplitude for target stimuli, and higher yet non-significant response in the shared condition, supporting increased arousal during the task.

The ERPs showed the expected heightened LPP responses to negative stimuli compared to neutral or positive ones^25,26,49^. However, shared attention had no effect on neural measures or on memory for emotional stimuli (as measured behaviorally). Indeed, previous work has yielded mixed results regarding the effects of emotional stimuli when manipulating the presence of others. While in some studies, participants reported enhanced feelings in the shared condition^6,12^, other studies found that shared experience tinted the subjective feelings more positive regardless of the stimulus valence (positive/ negative)^5^, reported an effect on behavioral measures (e.g., facial expressions) with no concurrent effect in self-reported emotions^17,18^, or did not find such an effect at all^14^. Our results echo the latter results and suggest that shared attention may have a more limited effect than previously suggested.

A potential account for the lack of shared attention effects may be due to the nature of the task itself. While we measured behavioral and neural differences in each valence condition, participants were not explicitly asked about their subjective feelings in response to the stimuli. Thus, we cannot rule out a potential shared attention effect on subjective emotional feelings, such as those reported by Wagner and colleagues^5^ and by Shteynberg *et al*.^12^. Thus, examining the relation between behavior, subjective “feelings” and neuronal responses is yet to be studied. An alternative explanation for the lack of an effect, is our choice of regions of interest, both in terms of EEG frequencies and ERP components. We chose a hypothesis driven approach, and specifically examined components based on prior research, using a sensor-driven approach. It is possible that a data-driven approach (e.g. component level analysis) would yield different results.

Our study had several methodological drawbacks that may limit the generalizability of its findings. While participants were recruited randomly in time slots of two, gender was not taken into account. This led to heterogeneity of the couples at the shared condition, as some of the couples were with the same gender and others were mixed. Although a recent study found no effect of gender on perception of IAPS pictures with social cues^50^, having couples with mixed gender may influence participants’ response to the stimuli^51,52^, may cause differences in arousal^53^ or create an implied in-group/out-group effect. Since the current study is underpowered to examine differences between same gender and mixed pairs, we encourage future studies to take this aspect under further investigation.

In addition, our only behavioral measure was accuracy rates, and reaction times (RTs) were not taken into account. It is possible that a shared attention effect, while missing from accuracy data, may still be revealed in RTs (or the occurrence of a speed-accuracy tradeoff).

In sum, our study provides a further examination of the role of shared attention on attention and recognition of emotional stimuli and suggests that it may be more restricted than previously described. We found no shared attention effects on memory for emotional stimuli, and no effect on relevant EEG measures of attention.

## Methods

### Participants

Forty-two undergraduate English-speaking students from the University of California Berkeley participated in the study. Participants’ age range was 18–38 years (*M* = 22.4 years, *SD* = 4.5), 22 participants were female, 6 were left-handed (self-reported) and 3 reported being ambidextrous. All participants reported normal or corrected to normal visual acuity and had no history of psychiatric or neurological disorders as confirmed by a screening interview. Participants received either course credit or payment for their participation and signed an informed consent to their participation. The study was approved by the local Ethics Committee (the University of California, Berkeley, Institutional Review Board) and was conducted in accordance with the Declaration of Helsinki.

### Study Design Overview

#### Experimental procedure

Participants enrolled to the experiment in slots of two and were instructed together. Participants completed 2 tasks: a flower counting task followed by a memory recognition task.

Flower counting task: The task was completed by each participant twice: both alone (“alone” condition) and concurrently with a partner (“shared” condition). Partners for the shared condition were assigned by study staff and did not know each other personally prior to the experiment. The order in which participants completed these two conditions was counterbalanced. During the flower counting task, EEG was recorded from participants in both the Alone and Shared conditions.

**Figure 4:**
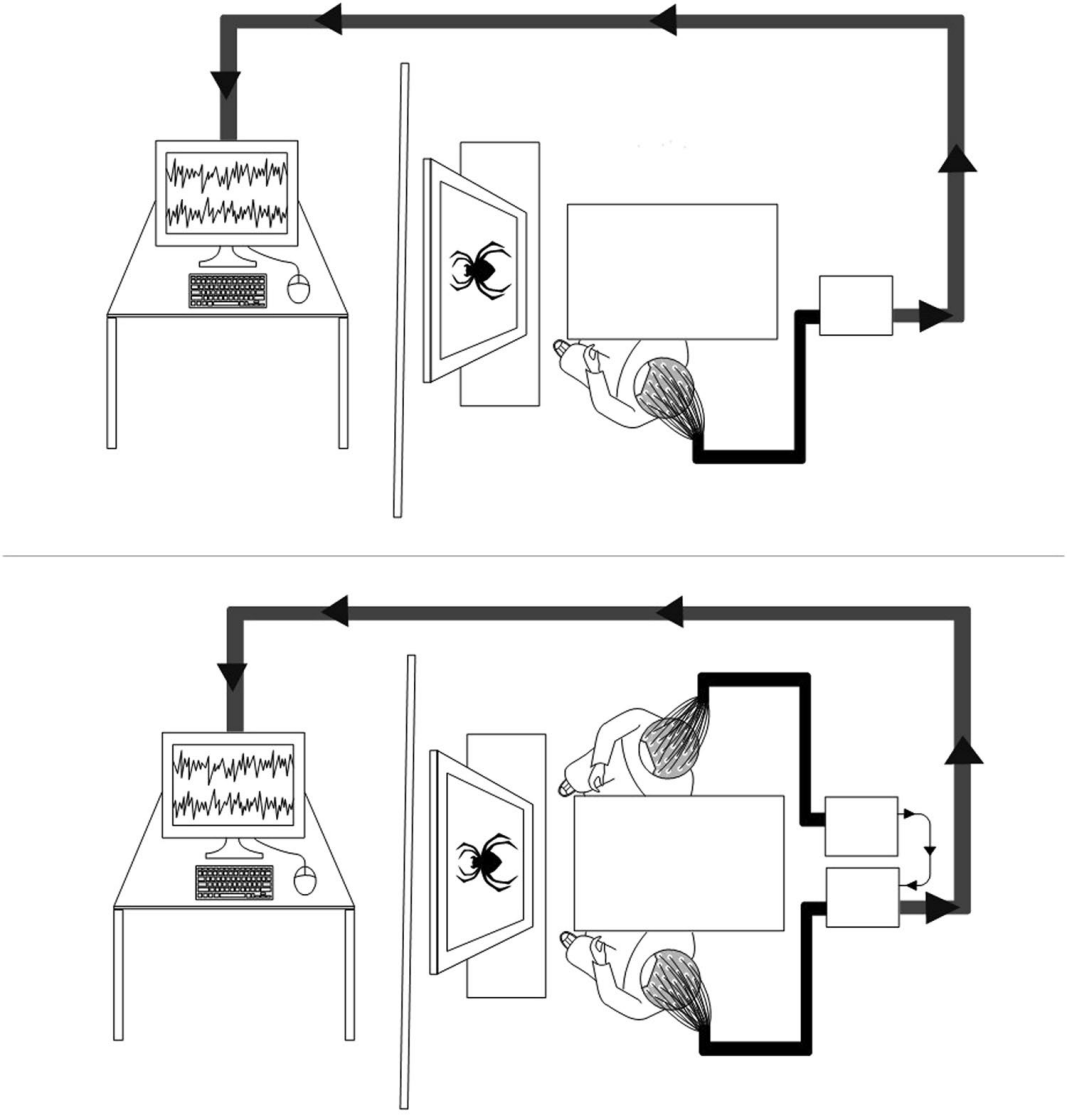
Experimental setup during performance of the flower counting task, in the alone (top) and shared (bottom) conditions. In the shared condition, a dual EEG setup was used, and amplifiers were synched using a ‘daisy-chained’ connection, which sent the information from both amplifiers to the experimental computer.

In the alone condition, one participant performed the flower counting task in the experiment room while the other subject sat in the waiting room. In the shared condition, the two participants performed the task simultaneously sitting in opposite sides of a table while viewing stimuli on a shared screen (see Fig. 4). At the completion of each block, participants wrote down the number of their counted flowers on a small piece of paper, which was then folded, without seeing or sharing their answers with one another. In this way, each participant repeated the task twice (alone- shared -wait, or wait- shared -alone), with different stimuli in each run. Each run lasted ~10 minutes and viewing parameters were kept constant for both conditions. The order of the runs, i.e. shared or alone, was counterbalanced between participants, such that if participant A did the task alone, and then together with participant B, B did the task first with A, then alone.

Recognition task: in the second part of the experiment, participants were placed in separate rooms and were asked to complete a memory recognition task. Participants were not told in advance that they will be required to memorize the stimuli, yet when instructed to perform the recognition task they were informed that the other participant (their partner in the shared condition) is also performing such a task in another room. The whole procedure took about 60 minutes.

#### Stimuli

We created three sets of 180 stimuli each taken from 540 unique pictures of the International Affective Picture System (IAPS;^16^). Each set was comprised of 60 negative, 60 positive and 60 neutral pictures. The three sets were counterbalanced for their valence (M ± SD; negative = 2.47 ± 1.53; positive = 7.22 ± 1.59; neutral = 5.02 ± 1.34) and arousal (M ± SD; negative = 5.81 ± 2.19; positive = 5.01 ± 2.26; neutral = 3.48 ± 1.99), using the published IAPS norms^16,22,23^. Two of the sets were used for the two repetitions of the flower counting task (see below), counterbalanced between subjects, while the third set, along with half of the images from each of the first two sets, was used for the recognition task (see below).

The target stimuli (flowers) were comprised of random pictures downloaded from the internet, approved for common use. Each picture depicted one flower. Note that emotional reactivity for these photos was not assessed, thus stimuli may have elicited a slightly positive emotional response in participants. Participants were not told why they were seeing emotional pictures during the task. All pictures were 9×12 cm in size.

### Behavioral Data Acquisition

#### Flower counting task

**Figure 5:**
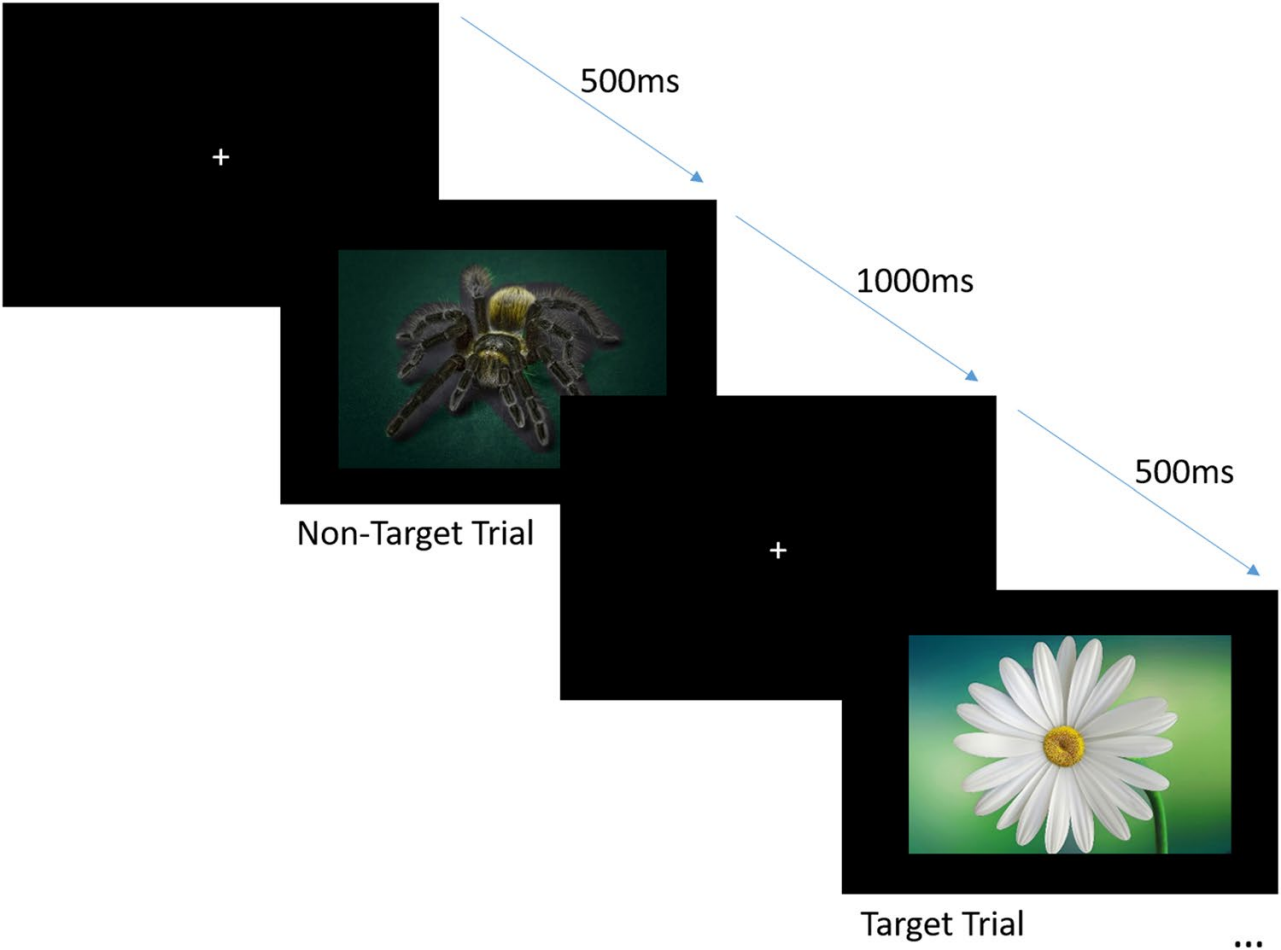
Trial procedure in the flower counting task. The sequence of trials in each block used in this task.

Each participant sat at a 45-degree angle, ~90 cm from a desktop screen (see Fig. 4). E-Prime 2.0 Professional software (Psychology Software Tools, Inc., Pittsburgh, PA) was used for stimulus presentation, using a Lenovo computer with a CRT monitor (ViewSonic P225f). On each trial, a fixation point was presented at the center of the screen for 500 ms, immediately followed by the stimulus, which was displayed for 1000 ms. Each stimulus contained either an IAPS picture (with equal probability for each stimulus type: neutral/negative/positive) or a flower picture, presented in a randomized order. The participant’s task was to silently count the number of flower pictures that appeared during the block and to write down their total number at the end of each block, a number which varied between 8 and 12. Participants completed two runs of this task (alone/shared), with each run comprised of 3 blocks of 60 IAPS pictures each and 8–12 target stimuli, lasting for up to 2 minutes. Participants were given the opportunity to rest in between blocks, hence a full run lasted ~10 minutes. Figure 4 has an illustration of the dual EEG setup and Fig. 5 depicts the experimental design.

#### Recognition task

Following the flower counting task, participants sat in separate rooms for a ‘surprise’ forced-choice recognition test. In order to test the effects of shared attention on recognition, we used a subset of 90 pictures from each of the two viewing conditions (alone/shared) and added a third set of 180 novel pictures that participants have not seen before, equated for valence and arousal to the first two sets, for a total of 360 IAPS pictures. Thus, the recognition task was comprised of 180 pictures that participants already saw during the flower counting task, and 180 novel pictures.

Participants were asked to decide, for each stimulus, whether they saw it during the flower counting task their performed earlier, by pressing one of two buttons presented on the screen (yes/no). Participants were given an unlimited time to respond. As soon as their response was recorded, the next stimulus appeared on the screen.

### EEG Data Acquisition

#### EEG recording

EEG recordings were performed during the flower counting task. EEG was recorded continuously from 64 Ag-AgCl pin-type active electrodes mounted on an elastic cap (Biosemi, http://www.biosemi.com/headcap.htm), according to the extended 10–20 system, and from two additional electrodes placed at the right and left mastoids. Data was recorded relative to CMS/DRL electrodes located between POz and PO3, while average voltage was kept in the range of ±40 mV signal using Biosemi’s electrodes offset tool. All electrodes were subsequently re-referenced digitally (see data processing below). Eye movements and blinks were monitored using bipolar horizontal and vertical Electro-oculography (EOG) derivations via two pairs of electrodes, with one pair attached to the external canthi and the other to the infraorbital and supraorbital regions of the right eye. Both EEG and EOG were digitally amplified and sampled at 1024 Hz using a Biosemi Active II system (www.biosemi.com). Triggers were sent to the Biosemi software and recorded along with the EEG data using a parallel port. When two participants were run together (Shared condition), one amplifier was ‘daisy-chained’ to the other, which then sent all information to the experimental computer. This ensured that the data received from both amplifiers was synchronized.

### Behavioral Data Analysis

For the flower counting task, we calculated the overall accuracy rate for each condition (alone/ shared). One participant had zero success rate in all blocks of the flower counting task and was hence excluded from analyses. Another participant was excluded due to missing data.

Accuracy rate was calculated as the ratio between the absolute distance between the number of counted flowers and the correct number of flowers, divided by the correct number of flowers in each experimental block, multiplied by 100. [**Accuracy rate** = 100 − (error *100), when **Error** (proportion) = the absolute distance (“how many I said there were” minus “how many were there”), **divided by the number of flowers** (= “how many were there”)].

For the recognition task, d’ was calculated as the difference between the number of hits (H; answering “yes” correctly) and false alarms (FA; answering “yes” incorrectly). Z-scores and sensitivity scores (d′ = z(H) − z(FA)) were derived in order to correct for the reported bias to say that emotional stimuli appeared^54,55^.

### EEG Data Analysis

#### EEG data processing

The EEG data was analyzed offline using the Brain Vision Analyzer software (Brain Products; www.brainproducts.com). Data was first filtered with a high-pass filter of 0.5 Hz and with a notch filter of 60 Hz (zero-phase shift IIR filter, 4^th^ order) and re-referenced to the common average activity from all electrodes. Individual noisy channels were elected using a semi-automated data inspection. Next, a low-pass filter at 30 Hz (zero-phase shift IIR filter, 4th order) was applied. Blinks and eye movement artifacts were identified and corrected using the Independent Component Analysis method (ICA infomax;^56^). Remaining EEG artifacts exceeding ±120 µV, with a voltage step of more than 50 µv/ms, activity under 0.5µv, or with a difference (max-min) of more than 150 µv were detected, and the data during an epoch of 300 ms symmetrically encompassing the event were excluded from the analysis.

#### Event related potential (ERP) analysis

The continuous, artefact-corrected EEG data was segmented separately for the two experimental conditions (alone/shared) and for each stimulus type (neutral/negative/positive/flower). Epochs were extracted for the appearance of the stimuli in windows of [−200 1000 ms], and the window of [−150:−50 ms] was used for baseline correction. Signals were averaged for each subject for each of the 8 resultant conditions (4 stimulus types in each of the 2 experimental conditions). In each block, the number of epochs was between 49–60 for each valence condition, and between 21–32 epochs for flower stimuli.

The Late-Positive Potential (LPP) was calculated for the neutral, negative and positive stimuli, as the mean activity in the 400–800 ms post-stimulus, in midline posterior-parietal electrodes (Pz CPz POz). This electrode combination is most frequently used to calculate LPP^27,57–62^. However, since some authors calculate LPP over a posterior-parietal electrode composition (C1, C2, CP1, CP2, Cz, CPz/Pz)^57,59,61–63^, we ran a second analysis using these electrode sites. We also performed a control analysis by calculating LPP mean activity in frontal sites (Fpz, Fp1, Fp2), to confirm that any observed differences between conditions were specific to the parietal LPP (see Supplementary Information).

The P3b was calculated for target stimuli (flowers) as the mean activity 350–600 ms post-stimulus, at the same posterior-parietal electrode locations (Pz CPz POz)^13,63^. We also ran a permutation analysis, each time iteratively (X 10000) randomly sampling (with return) 21 target and 21 Non-target items and recomputing test statistics.

#### Band power analysis

Alpha band power was computed for each participant, channel, and epoch. First, epochs were submitted to a Fast Fourier Transform (FFT; 0.5 Hz resolution, Hanning window within 10% overlap). Resulting amplitude values were squared to obtain power, averaged across frequency bins between 8 and 13 Hz, and normalized via log-transformation (log10). Mean alpha band power was subsequently averaged across segments in each experimental condition. We then transformed mean alpha power into a logarithmic scale, in order to normalize the data and dismiss any general individual changes in activity. Last, we averaged across occipital electrodes O1, O2 and Oz^43^ for alpha activity, and across central electrodes C3 and C4 for mu activity^31,37,38,45^.

### Statistical analysis

Statistical analyses were first performed using the statistical software package SPSS (IBM, version 20). Differences in accuracy rates of the flower counting task between conditions were analyzed using a paired Student’s t-test. Accuracy rates of the recognition task, d’ measures, alpha band values, and LPP activity values were all analyzed using a two-way repeated-measures Analysis of Variance (ANOVA) with a Greenhouse-Geiser correction with within-subject factors of condition (2 levels: alone and shared) and valence (3 levels: negative, neutral, positive). P3b values were analyzed using a two-way repeated-measures ANOVA with within-subject factors of condition (2 levels: alone and shared) and stimulus type (2 levels: target (flower) and non-target (neutral IAPS)). In all analyses, the Bonferroni correction was applied to correct for multiple comparisons. Results were considered significant at the level of p < 0.05.

To examine the strength of evidence to support null results, we further ran a Bayesian statistical analysis using the JASP software (Version 0.9.1)^64^, applying it for each of the previously described statistical tests. As we had no previous knowledge for prior probabilities, we referred to H_1_ and H_0_ as equal (Prior ratio = 1) and use the Bayes Factor (BF_10_) as our statistical measure in these tests^65–67^. Bayes Factor for interactions was extracted by the BF of the models with and without the interaction^68^.

Since the number of flowers and neutral stimuli were different, we further ran a permutation analysis for the P3b signal, using MATLAB (MathWorks, version R2018b). In order to create comparable sample sizes in each ERP calculation (the number of segments was 21–32 for flower events and between 53 to 60 for neutral IAPS events), we randomly sampled 21 segments with return from each stimulus type and repeated the procedure 10,000 times. The reported results correspond to the mode *F* across iterations, given degrees of freedom for the test (alpha level = 0.05; 95% CI).

## Supplementary information

Supplementary Information.

## Data Availability

The datasets generated during the current study are available from the corresponding author on reasonable request.
