# Supplementary material for: Behavioral and EEG Measures Show no Amplifying Effects of Shared Attention on Attention or Memory
Source: Sci Rep. 2020 May 21;10:8458. doi: 10.1038/s41598-020-65311-7 (PMC7242358; doi:10.1038/s41598-020-65311-7)
Supplement: Supplementary file 1 — Supplementary Information. [file 41598_2020_65311_MOESM1_ESM.pdf]

Behavioral and EEG Measures Show no Amplifying Effects of Shared Attention on Attention or Memory

Noam Mairon[1], Mor Nahum[1], Arjen Stolk[2], Robert T. Knight[2], Anat Perry[1,2*]

[1]The Hebrew University of Jerusalem, Jerusalem, Israel

[2]University of California, Berkeley, CA, USA

*Anat.perry@mail.huji.ac.il

Supplementary Information

P3b Results – Different Electrodes Distribution

Frontal Electrodes Distribution. We ran an additional P3b permutation analysis for frontal cites (Fp1, Fp2 and FPz) and found no effect of condition [$F(1,37) = 0.01$, $p = 0.91$, CI_F [0, 0.77]], effect of stimulus type [$F(1,37) = 35.36$, $p = 0.00$, CI_F [26.55, 46.57]] and no interaction [$F(1,37) = 0.03$, $p = 0.85$, CI_F [0, 2.12]].

LPP Results – Different Electrodes Distribution

Centro-Parietal Electrodes Distribution. As LPP is occasionally measured with a broader electrode range, we ran an additional analysis with Centro-parietal average (C1 C2, CP1, CP2, PZ, Cz, CPz, POZ): We conducted a 2X3 ANOVA [Condition X Valence (neutral/ negative/ positive)] for the LPP amplitudes recorded during the flower counting task. There was no significant effect of shared attention [$M \pm SD$ alone = .001 ± .13, shared = .12 ± .11; $F(1,37) = 1.8$, $p = .21$, $\eta p^2 = .048$, $BF_{10} = 0.63$]. Similarly to the analysis shown in the results section, we found increased neural LPP responses for stimuli with negative (low valence) compared to positive (high valence) or neutral [ERP amplitude neutral = .166 ± .09, negative = .48 ± .13, positive = .23 ± .12; $F(1.59,58.86) = 947$, $p = .001$, $\eta p^2 = .2$, $BF_{10} = 259.15$]. There was no significant interaction between Presence and Valence [$F(1.99,73.74) = 2.0$, $p = .14$, $\eta p^2 = .05$, $BF_{10} = 0.17$].

Frontal Electrodes Distribution. In order to see if this effect is specific to central-parietal cites, we ran an additional LPP analysis for frontal cites (Fp1, Fp2 and FPz): We conducted a 2X3 ANOVA [Condition X Valence (neutral/ negative/ positive)] for the LPP amplitudes. Similar to the central-parietal distribution, there was no

significant effect of shared attention [M ± SD alone = -1.84 ± .21, shared = -1.88 ± .17; $F(1,37) = .049$, $p = .82$, $\eta p^2 = .001$, $BF_{10} = 0.15$]. We also found decreased neural LPP responses for stimuli with negative compared to positive or neutral [ERP amplitude neutral = -1.59 ± .18, negative = -2.27 ± .22, positive = -1.72 ± .17; $F(1.77,65.73) = 10.52$, $p < .001$, $\eta p^2 = .22$, $BF_{10} = 523.93$]. There was no significant interaction between Presence and Valence [$F(1.96,72.44) = .25$, $p = .77$, $\eta p^2 = .007$, $BF_{10} = 0.09$].

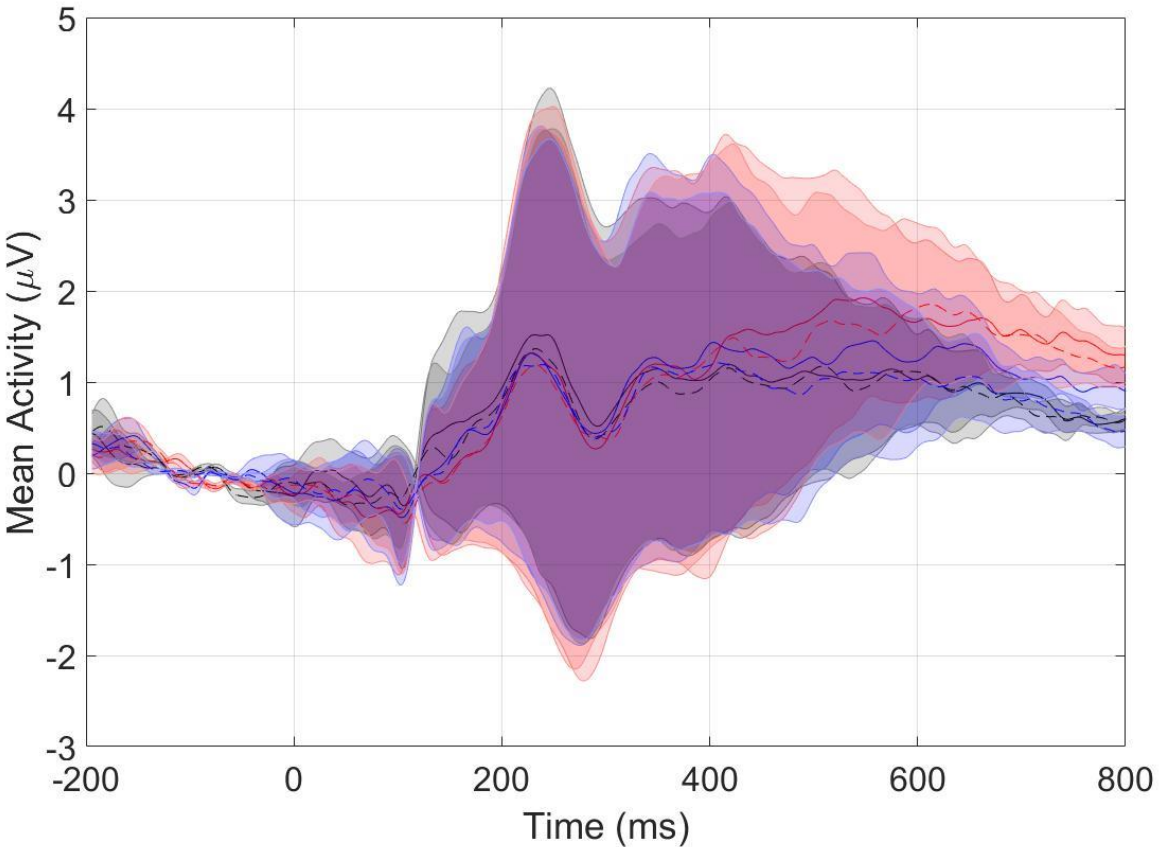

Supplementary Figure S1: LPP Results at Parietal cites. Mean ERP activity in the time window of [-100:800]ms (stimulus-locked) for POz, Pz and CPz, in alone (solid line) and shared (dashed line) conditions, for each stimulus valence: negative (red), positive (blue) and neutral (black). Shaded error regions represent standard deviation errors (using the ShadedErrorBar MATLAB function).
